# Successful Cæsarian Section after Death of Mother

**Published:** 1885-12

**Authors:** 


### SUCCESSFUL CÆSARIAN SECTION AFTER DEATH OF THE MOTHER.

DR. J. MACK HAYS, reports in the *New York Medical Record*, the following case :

Mrs. C., in good health, pregnant at eight and a half months, is suddenly seized with extensive cerebral congestion and dies of appoplexy soon after the Doctor arrives. The mother having felt motions shortly before her death, Dr. H. proposed to attempt to save the child, and the husband consenting, he proceeded without delay while life was still in the mother, to expose the uterus by a free incision, and as life was fast ebbing away, he incised the womb as low down as possible, sufficiently to admit two fingers. Using his fingers as a director, he slit up this organ to the placenta, evacuated the liquor amnii, and extracted a living child from the dead body of its mother. The author refers to *American Journal Medical Sciences*, Oct. 1879, *American Journal Obstetrics*, Jan. 1879, *British Medical Journal*, June 1879, &c., for similar cases.

